# Evaluation of the effectiveness of high-risk human papilloma self-sampling test for cervical cancer screening in Bolivia

**DOI:** 10.1186/s12879-020-04963-2

**Published:** 2020-04-03

**Authors:** Gustavo Allende, Pedro Surriabre, Neli Ovando, Pamela Calle, Andrea Torrico, Jaime Villarroel, Michel Bossens, Véronique Fontaine, Patricia Rodriguez

**Affiliations:** 1 Laboratory of Virology, Faculty of Medicine, Universidad Mayor de San Simón, Cochabamba, Bolivia; 2 Unité de Microbiologie Pharmaceutique et Hygiène, Faculté de Pharmacie, Université Libre de Bruxelles (ULB), CP205/2, Campus Plaine, Boulevard du Triomphe, 1050 Brussels, Belgium; 3 Anatomopathology Laboratory of the Bolivian-Japanese hospital, Cochabamba, Bolivia; 4 Research Laboratory in Human Reproduction, Campus Erasme, Faculty of Medicine, Universite libre de Bruxelles, Brussels, ULB Belgium

**Keywords:** Cervical intra-epithelial neoplasia, Cytology, Human papillomavirus testing, visual inspection with acetic acid

## Abstract

**Background:**

In Bolivia the incidence and mortality rates of uterine cervix cancer are the highest in America. The main factor contributing to this situation is the difficulty of establishing and maintaining quality prevention programs based on cytology. We aimed to evaluate the effectiveness of HR-HPV testing on self-collected samples to detect cervical intra-epithelial neoplasia and identify the best combination of screening tests.

**Methods:**

A total of 469 women, divided in two groups, were included in this study. The first group included 362 women that underwent three consecutively primary screening tests: self-collected sampling for HR-HPV detection, conventional cervical cytology and visual inspection under acetic acid (VIA). The second group included 107 women referred with a positive HR-HPV test that underwent conventional cervical cytology and VIA. The presence of high grade intraepithelial lesion (CIN 2+) or invasive cancer was verified by colposcopy and biopsy.

**Result:**

In the screening group the sensitivity to detect high grade intraepithelial lesion (CIN 2+) or invasive cancer were 100, 76, 44% for the VIA, HR-HPV test and cytology, respectively. In the referred group, the sensitivity to detect high grade intraepithelial lesion (CIN 2+) or invasive cancer by VIA and cytology were 100 and 81%, respectively.

**Conclusions:**

VIA and HR-HPV self-sampling were the best combination to detect CIN2+ lesions. Cytology analysis gave the poorest performance.

## Background

Worldwide, cervical cancer is the fourth leading cause of death among women [1]. In 2018, 570,000 new cases of cervical cancer were diagnosed worldwide; the vast majority, around 90%, occurred in low income countries [1]. Screening program based on cytology in developed countries has been proven successful, resulting in a rapid decrease in the incidence and mortality rates of cervical cancer [2]. In developing countries, the incidence of cervical cancer remains high. The main factor contributing to this situation is the difficulty to establish and maintain quality prevention programs based on cytology (Papanicolaou). This could explain partially the low screening coverage [3]. To improve the screening in developing countries, other screening methods including the high-risk human papilloma virus (HR-HPV) DNA detection were introduced [4]. The HPV tests are based on the identification of persistent infections by HR-HPV that are essential for the development of precancerous cervical lesions and cervical cancer [4, 5]. The use of this method as a screening test for cervical high-grade intraepithelial neoplasia (CIN 2 +), turned out to be highly effective, in term of sensitivity, and could be an alternative in cervical cancer primary screening [6]. Another advantage is that it allows self-sampling, an interesting procedure in rural areas, as it removed barriers related to clinic visit apprehension and the discomfort of a cytological test [7] .On the other hand, visual inspection with acetic acid (VIA) is also an effective test for the prevention of cervical cancer, allowing detection of early cervical epithelia changes after the acetic acid (3–5%) application for the prevention of cervical cancer in low-resource settings [8, 9]. However, its clinical performance is observer dependent [9].

The situation in Bolivia is alarming regarding the incidence and mortality rates of uterine cervix cancer, with the highest incidence in America (38 per 100,000 women) and a mortality rate of 19 per 100,000 women (rates standardized by age) [10]. The main reasons to inadequate prevention of cervical cancer in Bolivia are the low screening coverage of the Papanicolaou smear cytology test (Pap smear), the lack of follow up of the positive cases, the poor information given on cervical cancer prevention, the deficient training of the health staff, the low credibility in the public health system and finally economic, geographic and sociocultural barriers reducing the access of the Bolivian to health care centers [11, 12].

Screening, offered by the first level of care, responsible for prevention, is available free of charge, for sexually active women until 64 years old [13]. Nevertheless, Pap smear coverage, from 2005 to 2016, didn’t exceed 16.6% and coverage of VIA in 2015 and 2016 didn’t exceed 19% [14]. The health personnel estimated that 50 to 80% of PAP screened women were lost to follow-up, mainly because of delays in result delivery [11].

To improve this situation, our group has recently evaluated the acceptability of introducing an HR-HPV test on self-collected samples with cheap and simple devices [12, 15]. Indeed, we have previously shown the analytical performance of the HR-HPV detection on samples self-collected with a cotton swab, dried before transport on glass slide [15]. Our HR-HPV detection method involves two PCR based techniques: a BSGP5+/6+ PCR–EIA method and a pU1ML-2R PCR. This strategy was recently described and analytically validated in comparison to the commercially available Hybrid Capture II method [16].

The purpose of this study is to determine the clinical effectiveness of our HR-HPV DNA detection strategy on self-collected samples to detect high grade intraepithelial lesions and cervical cancers, comparing it to Pap smear analysis and VIA, in order to identify the best combination to screen Bolivian women or to sort women at risk.

## Methods

### Study population

Between January 2016 and April 2018, a cross-sectional study was carried out in Cochabamba (Bolivia) within the framework of the inter-institutional cooperation agreement between the “Universidad Mayor de San Simon” and the Public health service of Cochabamba (SEDES). Through these two institutions, a cervical pathology clinic was equipped at Hospital Cochabamba, where the present study was carried out. The staff consisted of a nurse assistant and a gynecologist trained in colposcopy and VIA during the two previous years.

The Bio-ethical committee of the “Universidad Mayor de San Simon” approved the study protocol (December 12, 2015). A total of 469 women were included in this study, divided in two groups.

**Fig. 1:**
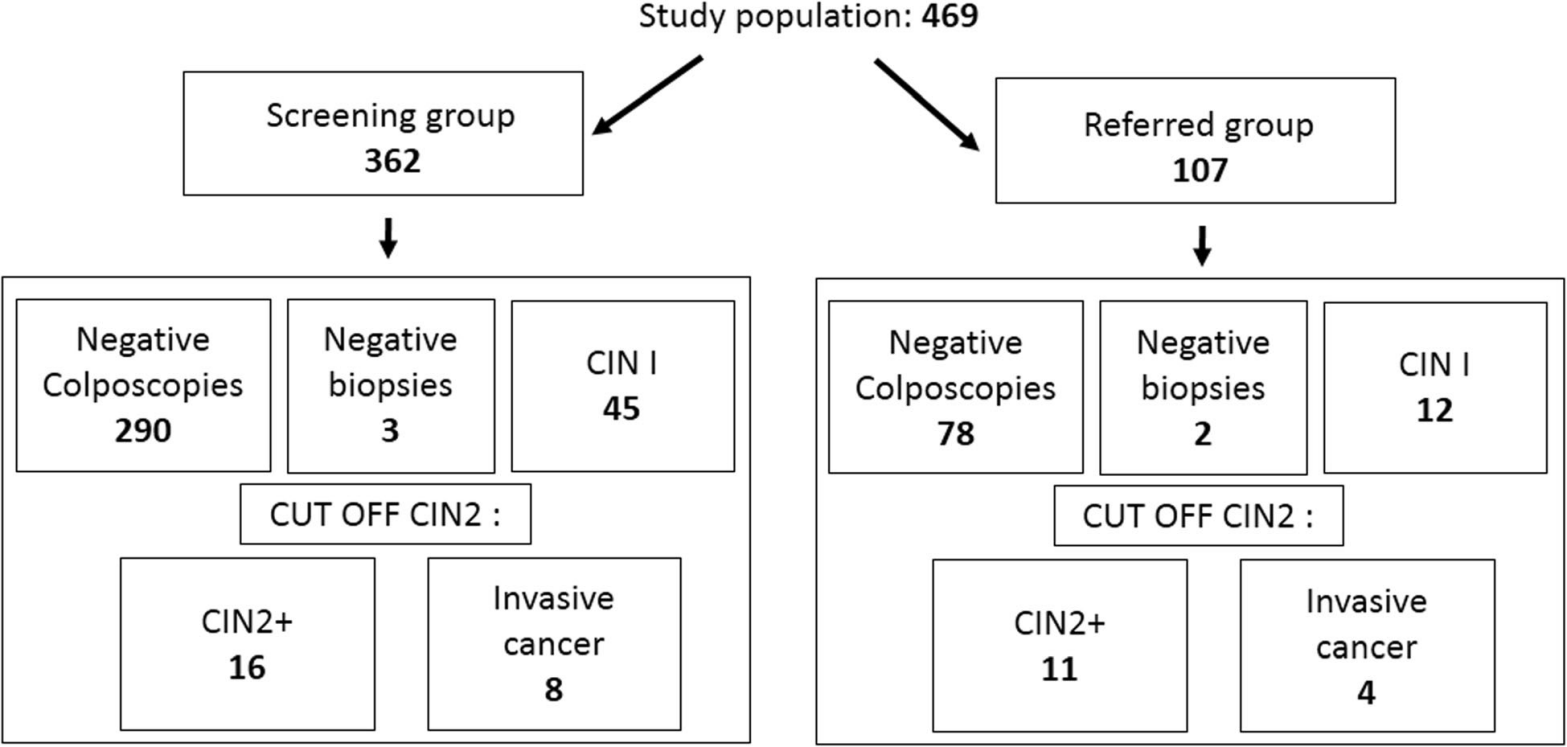
Colposcopic and biopsy results in the screening and referred groups. The number of study participants is given in bold

The first group (phase 1) included 362 women that underwent three screening tests: HR-HPV DNA detection, Pap smear, and VIA, performed consecutively at the consultation. The women in this group assisted spontaneously or were referred from general medicine clinics to the cervical pathology clinic of Cochabamba hospital due to their gynecological symptoms. This phase 1 study was named “the screening group”. The second group (phase 2) included 107 women referred with a positive HR -HPV DNA detection test, these women were identified in cervical cancer prevention campaigns conducted in different geographical areas of the city of Cochabamba (urban and rural). They underwent additional Pap smear and VIA tests, and this study group was named “the referred group”. All women (phase 1 and 2) were examined by colposcopy after the three (screening group) or two (referred group) screening tests. Biopsy was performed when necessary, according to the colposcopy observation results. (Fig. 1). In addition, data on the characteristics of the population were collected from the gynecological clinical history and from our own project database.

The signed informed consent and an adequate colposcopy were indispensable requisites for the patient inclusion in this study. Pregnant women and women with a hysterectomy or under 25 years of age or older than 64 years were excluded from this study according to nationals and international recommendations. In the referred group an additional inclusion factor was applied as all participants were required to have a positive HR-HPV test. In contrast, we applied an additional exclusion factor for the screening group, being that none of patients should have had previous results, positive or negative, of a HR-HPV test, VIA test or cytology analysis. All inclusion and exclusion factors were applied before the gynecological evaluation, except for the “adequate colposcopy” inclusion factor that was applied during the colposcopy evaluation.

### HR-HPV detection test

A kit, previously described and analytically validated, was provided to the women at the clinic consultation (or, for the referred group women, eventually at home or on the marked during promotional campaigns) to perform vaginal self-sampling in order to analyze their samples in a HR-HPV DNA detection assay [15]. The kit contained a pair of sterile gloves, a cotton swab and a sterile glass slide fixed in a paper box on which the personal data of the patient can be written [15]. The protocol for the use of the self-sampling device was explained in detail to the woman by a trained health staff for both groups (screening and referred group) as previously described [15]. The extracted DNA quality was first evaluated by a ß-globin PCR. Only samples with ß-globin PCR positive results were further processed. Two consensus PCR (BSGP5+/6+ and PU1ML-2R) allowed for HR-HPV detection [16]. The BSGP5+/6+ primers are an improvement of the original set of primers GP5+/6+ targeting the HPV L1 sequences [16] An immune-enzymatic assay (EIA) was performed subsequently on BSGP5+/6+ PCR samples to identify specifically HR-HPV types 16, 18, 31, 33, 35, 39, 45, 51, 52, 56, 58, 59 using digoxigenin-labelled probes [16]. The pU1ML-2R PCR targets the E6/E7 regions of at least 6 HR-HPV types: 16, 18, 31, 33, 52 and 58. A sample was considered positive for HR-HPV when any of these two tests (PU1ML-2R PCR and BSGP5+/6+ PCR/EIA) yielded a positive result [16].

### Cytological analysis

Samples for conventional cytology analysis (Pap smear) were taken from the cervical transformation zone by the gynecologist. The smear was spread on a glass slide and stained, according to the Papanicolaou method [17]. The analyses were performed at the Viedma Hospital cytology laboratory and in private cytology laboratories, as requested by the patients. The quality control of public and private cytopathology laboratories is carried out annually under the supervision of the Ministry of Health of Bolivia through the “Normative Laboratory Documents” [18]. A cytology test was considered positive in case of ASCUS or more severe lesion.

### Visual inspection under acetic acid

The Visual Inspection under Acetic Acid (VIA) was made with white wine vinegar with an acetic acid concentration of 5%, one minute after the application. The solution was changed every 2 weeks. The cervix was observed under a white LED light and the test was considered positive when the presence of aceto-white lesions was found in the squamo-columnar junction or near to an opening in the center of the ectocervix known as Os. Otherwise it was considered negative [19]. VIA results were recorded in a notebook by a nurse before colposcopy analysis.

### Colposcopy

Colposcopy was a separate independent examination, performed after the three screening tests, on the same day (VIA and colposcopy were done by the same examiner). The decision to take a biopsy was based on the results of the colposcopy, using the classification of the IFCPC (“Adequate/inadequate” “normal “, “minor lesion or colposcopy grade I”, major lesion or colposcopy grade II”,” and “suspicious for invasion”) [20]. A biopsy was taken when the results of the colposcopy was equal or greater than a colposcopy grade I (minor lesion), The diagnosis of a cervix without anomalies was provided by the colposcopic impression, in these cases the biopsy was no taken, according to Bolivian guidelines [21].

### Biopsy analysis

Biopsies were processed in the reference laboratory of Pathology of the Bolivian-Japanese gastroenterological hospital. Histological classification (CIN) of epithelial lesions of the cervix were used according to the description made by Richart [22]. Samples of 29 biopsies were chosen by simple random sampling out of 101 biopsies between low-grade (CIN1), high-grade intraepithelial lesions (CIN2, CIN3) and invasive cancer were sent for quality control to the laboratory of Prof. Philippe Delvenne (Department of Pathological Anatomy, B23 CHU Sart Tilman - 4000 Liège, Belgium) for rereading.

A CIN 2 severity grade was determined as the “cut off” in the biopsy result in this study, considering that people whose lesion were exceeding this cut off had a positive gold standard result while those having a lower severity grade results were considered as negative in this gold standard assay.

### Statistics

Statistical analyses were performed independently on available results using cross tables to determine the sensitivity, specificity and predictive values of each test. Number of true positives, true negative, false positives and false negative cases were calculated considering the biopsy analysis result (obtained in Bolivia) of CIN2 as a cut off to detect CIN2+ and cervical cancer (gold standard for positive cases) and considering negative colposcopy result or normal or CIN1 biopsy analysis result as negative cases. The confidence limits for each proportion were calculated with OpenEpi software (Normal Approx.)**.**

## Results

### Characteristics of the study population

Characteristics of the women, aged between 25 and 64 years, participating either to the screening group (*n* = 362) or the referred group (*n* = 107) are presented in Table 1.

**Table 1:** Characteristics of the population found in the two groups

	Screening (362) N			Referred (107) N			Screening and Referred (469) N		
	Data(n)	mean	No data(n)	Data(n)	mean	No data(n)	Data(n)	mean	No data(n)
Age	362	40	0	107	36.2	0	469	38.1	0
Menarche	335	13	27	68	12.9	39	403	12.95	66
First sexual intercourse	335	18.4	27	68	17.5	39	403	17.95	66
Number of sexual partners	334	2	28	68	2.2	39	402	2.1	67
Pregnancies	320	3	42	67	2.9	40	387	2.95	82

### Sensitivity, specificity and predictive values of HR- HPV, pap smear and VIA to detect CIN 2+ or worse lesions in a primary screened population

In the first phase 1 of our study we compared three primary screening tests to detect high-grade intraepithelial lesions and cervical cancer (CIN2+ or worse) in our study population. This phase 1 study, was performed on 362 women, named “the screening group”, that underwent, consecutively, HR-HPV DNA detection by self-sampling, Pap smear and VIA, before colposcopy. A biopsy was performed when necessary, according to the colposcopy observation results. In this screened group, 72 biopsies were taken, out of which16 had high-grade intraepithelial lesions (CIN2+) and 8 had invasive cancer. The rest of the biopsies had a result lower than CIN2. The sensitivity, specificity and predictive values of the three screening tests to detect CIN2+ lesions or worse, based on the biopsy results (gold standard) were determined through the analysis of the results of 362 patients. The sensitivity and specificity were respectively: 44 and 93% for the PAP, 100 and 86% for the VIA and 76 and 78% for the HR-HPV test. The positive and negative predictive values were, respectively, 30 and 96% for PAP, 35 and 100% for VIA, 18 and 98% for HR-HPV test (Table 2).

**Table 2:** Screening group (CIN 2+ or worse)

**Cytology**^a^	N	Negative reference test	Positive reference test	PPV %	NPV %	95% IC
Positive	26	18	8	30		13–48
Negative	257	247	10		96	93–98
Total	283					
Sensitivity			44%			21–67
Specificity		93%				90–96
**VIA**		Negative reference test	Positive reference test	PPV %	NPV %	95% IC
Positive	68	44	24	35		24–46
Negative	294	294	0		100	
Total	362					
Sensitivity			100%			
Specificity		86%				83–90
**HPV**^b^	N	Negative reference test	Positive reference test	PPV %	NPV %	95% IC
Positive	87	71	16	18		10–26
Negative	259	254	5		98	96–99
total	346					
Sensitivity			76%			57–94
Specificity		78%				73–82

^a^cytology lost samples = 79

^b^HPV lost samples = 16

### Sensitivity, specificity and predictive values of pap smear and VIA to detect CIN 2+ or worse lesion in a HR-HPV DNA positive population

In the group of referred women, 29 biopsies were obtained, from which 11 corresponded to high-grade intraepithelial lesions (CIN2+) and 4 to invasive cancers, the rest of the biopsies had a result lower than CIN2. The sensitivity, specificity and positive values of the two screening tests to detect high-grade intraepithelial lesions and cervical cancer (CIN2+ or worse) were determined through the analysis of the results of 107 patients. The sensitivity and specificity were respectively: 81 and 88% for the PAP, 100 and 85% for the VIA and the positive and negative predictive values were 64 and 95% for the PAP, 53 and 100% for the VIA, respectively (Table 3).

**Table 3:** Referred group CIN2+ or worse

Cytology^a^	N	Negative reference test	Positive reference test	PPV %	NPV %	95% IC
Positive	14	5	9	64		39–89
Negative	42	40	2		95	89–100
Total	56					
Sensitivity			81%			59–100
Specificity		88%				79–98
VIA	N	Negative reference test	Positive reference test	PPV %	NPV %	95% IC
Positive	28	13	15	53		35–72
Negative	79	79	0		100	
Total	107					
Sensitivity			100%			
Specificity		85%				78–92

^a^lost samples: 51

### Quality control of the gold standard procedure

The Bolivian anatomo-pathology analyses were assessed by rereading in a Belgian laboratory, 29 biopsies out of 101 biopsies, chosen randomly by a technician from the Laboratory of Virology at the Universidad Mayor de San Simón, Cochabamba, Bolivia in the case of CIN1 lesion biopsies or according to availabilities for the CIN2+ lesion biopsies. The comparison of biopsies results between the Bolivian anatomo-pathology reference laboratory and the CHU Liège pathology laboratory (Belgium) gave a concordance of 84% for the CIN2 + lesions and an agreement of 70% for the lesions CIN 1 lesions (Additional file 1).

### Screening samples lost in the study population

We analyzed sample lost in the two study group. There were losses in the results of the HR-HPV test (4% in the screened group) but also mainly in the cytology analyses (22 and 48% in the screened and referred group, respectively). As VIA was performed immediately, on the place, all the results were available for this test (Table 4).

**Table 4:** Lost samples in the study population

Screening group	Study population N (%)	Lost samples N (%)	Final Study population N (%)
Cytology	362 (100)	79 (22)	283 (78)
VIA	362 (100)	0	362 (100)
HPV	362/100)	16 (4)	346 (96)
**Referred group**			
Cytology	107 (100)	51 (48)	56 (52)
VIA	107 (100)	0	107 (100)

## Discussion

Bolivia has one of the highest levels of incidence and mortality of cervical cancer in America [10]. This study was conducted to compare the effectiveness to detect CIN 2+ or worse lesions using either one of the two primary screening tests usually performed in Bolivia, the Pap cytological test and the visual inspection with acetic acid test, or the HR-HPV DNA test, performed here by a new PCR/EIA based strategy on self-collected samples. Indeed, we previously reported that the PCR/EIA strategy had a very good performance compared to the hybrid capture II test (kappa value of 0.82) [16].

The correct performance of a screening test can be assessed by analyzing several characteristics: the diagnostic accuracy to discriminate between a target condition and a good health, measured by the sensitivity and specificity values, and, on the other hand, the positive and negative predictive values to evaluate the performance of a screening test in a population [23].

The clinic sensitivity and specificity of our low cost self-sample HR-HPV test to detect CIN 2+ or worse lesions in the screened group were 76 and 78%, respectively, giving higher detection values than cytology but lower to the VIA. Despite its moderate sensitivity to detect a CIN2+ lesion or worse, its negative predictive value was high as expected (98%). It is worth noting that the HR-HPV test was robust, giving results with no inter-observer variation [16]. The main advantage of this screening method is that it allows reaching women from rural areas or women rejecting the gynecological revision [7, 12, 15, 24]. As the VIA test, the HPV test had a low positive predictive value (18%) requesting an additional triage methodology, as previously reported [25–29].

The cytology in the screened group showed a low sensitivity (21–67%) and a specificity of 92%, to detect CIN2 + lesion or worse, giving the lowest performance amongst the evaluated tests. Similar results were obtained in an extensive study conducted in European and North American countries, reporting a low sensitivity (53%) and a high specificity (96%) of the PAP staining to detect CIN2 + lesions [6]. The poor performance of the cytology in developing countries and the poor follow-up of positive cases led to the introduction of alternative screening approaches, based on VIA and/or on HR-HPV detection tests.

The screening of patients with VIA allowed to detect high-grade intraepithelial lesions (CIN2 + or worse) with 100% sensitivity and 86% specificity. Other studies reported similar results with 79% sensitivity and 89% specificity in Africa and India [30], 83% sensitivity and 89% specificity in Mongolia [31], 73% sensitivity and 80% specificity in Kenya [32]. Nevertheless other studies obtained contrasting results with a low sensitivity and high specificity in Rwanda (41% Sensitivity and 96% specificity), [33] or a moderate sensitivity with high specificity in Thailand (67.9% sensitivity and 100% specificity) [34]. This considerable variation between studies on the accuracy of VIA in the detection of high-grade lesions (CIN2 +) is possibly attributable to inter-observer variation, skill levels, light used and acetic acid concentration [35] .In our study, as there was only one observer previously trained in visual screening and colposcopy (2 years) that could explain the 100% sensitivity.

VIA generally gave a large number of false positive, leading to low positive predictive value (34%) [36] .This indicates the need of a triage method to avoid a burden to public health-care service by a large number of required confirmatory colposcopies under gynecological review. This is especially important in Bolivia as the public health system has great limitations for the follow-up and treatment of patients [11].

We also assessed the performance of the VIA and the Pap smear test as triage method for HR-HPV positive women (referred women). Although the number of samples with CIN2 + lesions was small, because losses of samples in cytology (around 50%), the results suggested that both tests give better positive predicted value in triage compared to their use in screening.

Bolivia is one of the poorest countries in South America and its public health system had difficulties in the monitoring and treatment of pre-malignant lesions of the cervix detected by cytology. This is partly due to the fact that 38% of the cytology results were lost [37], as observed in our study, with a loss of 22 and 48% of the cytological results in the screening group and referred group respectively. Furthermore, cytological results were delivered only after a minimal 45 days. Taking this into account but also the low sensitivity found for cytological analyses, many women were not being properly diagnosed with CIN2+ or cervical cancer (67% of the screening group and 40% of the referred group) when diagnosis was only based on cytology. On the other hand only some health services have colposcopes and few personnel are trained to treat pre malignant lesions [11, 13]. In that context, the strategy of a “unique visit” to “see and treat” in which treatment by cryotherapy or cold coagulation is eventually provided to women with positive detection results in the HR-HPV detection test and VIA analysis, is particularly adapted for low-income countries [38, 39]. This strategy could be the most suitable for Bolivia to reduce the incidence and mortality of cervical cancer. Furthermore, HPV based screening could allow extension of the cervical screening interval beyond 5 years, potentially reducing the public health burden, as recently proposed [40].

A limiting factor in our study was that a confirmatory biopsy was not performed on women with a normal colposcopy result. The final result on the condition of their cervix was based only on colposcopy impression (that could have led to the higher specificity of the VIA). This limitation was due to ethical and technical reasons, as our intervention followed the official guidelines from the Bolivian Ministry of Health [21]. This could have also reduced the specificity of the cytology and HR-HPV tests (by increasing the amount of false positives). Indeed, there could be microscopic CIN2 + lesions not visible to the human eye or by colposcopy examination, but that still could be detected by cytology or the HR-HPV test, which would have led to false categorization, as false positives. Indeed colposcopy is not sufficiently precise, although useful in estimating lesion grade [41–44]. Nevertheless, most studies, like us, determine the final negative status of the cervix for CIN 2 through colposcopy when lesion is not evident, and use this result as a true negative clinical condition [32, 45–47].

## Conclusions

Our results suggest that the VIA and our low cost HR-HPV DNA PCR detection assay have better sensitivity to detect cervical cancer and its true precursor lesions, compared to standard Bolivian PAP smear test. Among all evaluated test the cytology analysis showed the lowest performance to detect CIN 2+ lesion or worse, even at the lowest cytological cut-off (ASCUS).

Since our self-sampled HR-HPV detection test (HPV screening) could overcome sociocultural barriers and to increase screening coverage [12], an also showed an acceptable sensitivity and negative predictive value, we propose that it could be combined with a triage method, like the VIA (i.e. in a see and treat strategy), considering the complementary high specificity and the possibility to give immediate results with the VIA, to eventually immediately allow for CIN2+ lesion treatment by cryotherapy. The cytology analysis, Pap smear test, on the other hand, is not the most appropriate screening test for Bolivia due to its poor performance to detect CIN2 + or worse, its long delay for delivering result and its large number of lost samples.

Finally, to improve the screening coverage, it is also essential to better sensitize the population through the education and facilitate access to screening results. Furthermore, it is essential to establish monitoring of the flow of exams, to identify the cause of delaying results and to develop new strategies that could insure delivery of the results in time but also that could improve the patient adherence to their gynecological health follow-up.

## Supplementary information

**Additional file 1.** Bolivian anatomo-pathology analyses, by rereading 29 biopsies out of 101 biopsies chosen at random in a Belgian laboratory.

## Data Availability

The datasets used and/or analyzed during the current study are de-identified and available from the corresponding author on reasonable request.

## Abbreviations

HPV: Human papillomavirus
DNA: Deoxyribonucleic acid
PCR: Polymerase chain reaction
HR-HPV: High-risk human papillomavirus
VIA:Visual: Inspection with Acetic Acid
CIN: Cervical Intraepithelial Neoplasia
PPV: Positive predictive value
NPV: Negative predictive value

## References

[1] WHO | Cervical cancer, Accessed 23 Jan 2019. http://www.who.int/cancer/prevention/diagnosis-screening/cervical-cancer/en/.

[2] Anttila A, Pukkala E, Söderman B, Kallio M, Nieminen P, Hakama M (1999). Effect of organised screening on cervical cancer incidence and mortality in Finland, 1963-1995: recent increase in cervical cancer incidence. Int J Cancer.

[3] Sankaranarayanan R, Budukh AM, Rajkumar R (2001). Effective screening programmes for cervical cancer in low- and middle-income developing countries. Bull World Health Organ.

[4] zur Hausen H (2009). Papillomaviruses in the causation of human cancers - a brief historical account. Virology.

[5] Walboomers JMM, Jacobs MV, Manos MM, Bosch FX, Kummer JA, Shah KV (1999). Human papillomavirus is a necessary cause of invasive cervical cancer worldwide. J Pathol.

[6] Cuzick J, Clavel C, Petry K-U, Meijer CJLM, Hoyer H, Ratnam S (2006). Overview of the European and north American studies on HPV testing in primary cervical cancer screening. Int J Cancer.

[7] Gupta S, Palmer C, Bik EM, Cardenas JP, Nuñez H, Kraal L (2018). Self-Sampling for Human Papillomavirus Testing: Increased Cervical Cancer Screening Participation and Incorporation in International Screening Programs. Front Public Health.

[8] Nooh AM, Mohamed ME-S, El-Alfy Y (2015). Visual inspection of cervix with acetic acid as a screening modality for cervical Cancer. J Low Genit Tract Dis.

[9] World Health Organization, World Health Organization, Reproductive Health and Research. Comprehensive cervical cancer control: a guide to essential practice. 2014. http://apps.who.int/iris/bitstream/10665/144785/1/9789241548953_eng.pdf?ua=1. Accessed 26 Mar 2018. 25642554

[10] Cancer today. https://gco.iarc.fr/today/data/factsheets/populations/68-bolivia-plurinational-state-of-fact-sheets.pdf. Accessed 12 Feb 2019.

[11] Dzuba IG, Calderón R, Bliesner S, Luciani S, Amado F, Jacob M (2005). A participatory assessment to identify strategies for improved cervical cancer prevention and treatment in Bolivia. Rev Panam Salud Publica.

[12] Allende G, Surriabre P, Cáceres L, Bellot D, Ovando N, Torrico A (2019). Evaluation of the self-sampling for cervical cancer screening in Bolivia. BMC Public Health.

[13] Estado Plurinacional de Bolivia Ministerio de Salud Y deporte. Plan nacional de prevención control y seguimiento de cáncer de cuello uterino 2009–2015 https://www.iccp-portal.org/system/files/plans/plan_cancer_cuello_uterino.pdf. Accessed 23 Jan 2019.

[14] Documentos CAI Nacional 2016. http://snis.minsalud.gob.bo/publicaciones/category/91-documentos-cai-nacional-2016. Accessed 25 Mar 2018.

[15] Surriabre P, Allende G, Prado M, Cáceres L, Bellot D, Torrico A (2017). Self-sampling for human papillomavirus DNA detection: a preliminary study of compliance and feasibility in BOLIVIA. BMC Womens Health.

[16] Surriabre P, Torrico A, Vargas T, Ugarte F, Rodriguez P, Fontaine V (2019). Assessment of a new low-cost, PCR-based strategy for high-risk human papillomavirus DNA detection for cervical cancer prevention. BMC Infect Dis.

[17] THE BETHESDA SYSTEM. http://screening.iarc.fr/atlasclassifbethesda.php. Accessed 12 Nov 2018.

[18] Ministerio de Salud, Estado plurinacional de Bolivia. Documentos Normativos de Laboratorio https://www.minsalud.gob.bo/programas-de-salud/coordinacion-nacional-de-laboratorios. Accessed 28 Nov 2019.

[19] Sankaranarayanan R, Wesley RS (2003). International Agency for Research on Cancer. A practical manual on visual screening for cervical neoplasia.

[20] Nam K (2018). Colposcopy at a turning point. Obstet Gynecol Sci.

[21] Estado plurinacional de Bolivia M de S y *D. Norma* Nacional,reglas, protocolos y procedimientos para la deteccion del cancer de cuello uterino. 2009. https://www.minsalud.gob.bo/images/Documentacion/redes_salud/NORMA%20NACIONAL%20REGLAS%20PROTOCOLOS%20Y%20PROCEDMIENTOS%20PARA%20LA%20DET.pdf.

[22] Richart RM (1973). Cervical intraepithelial neoplasia. Pathol Annu.

[23] Šimundić A-M (2009). Measures of diagnostic accuracy: basic definitions. EJIFCC.

[24] Broquet C, Triboullier D, Untiet S, Schafer S, Petignat P, Vassilakos P (2015). Acceptability of self-collected vaginal samples for HPV testing in an urban and rural population of Madagascar. Afr Health Sci.

[25] Wentzensen N, Schiffman M, Palmer T, Arbyn M (2016). Triage of HPV positive women in cervical cancer screening. J Clin Virol.

[26] Isidean SD, Mayrand M-H, Ramanakumar AV, Rodrigues I, Ferenczy A, Ratnam S (2017). Comparison of triage strategies for HPV-positive women: Canadian cervical Cancer screening trial results. Cancer Epidemiol Biomark Prev.

[27] Mishra GA, Pimple SA, Gupta SD (2019). Cervical Cancer screening in low resource settings: cytology versus HPV triage for VIA positive women. Int J Prev Med.

[28] Benski A-C, Viviano M, Jinoro J, Alec M, Catarino R, Herniainasolo J (2019). HPV self-testing for primary cervical cancer screening in Madagascar: VIA/VILI triage compliance in HPV-positive women. PLoS One.

[29] Lazcano-Ponce E, Lorincz AT, Cruz-Valdez A, Salmerón J, Uribe P, Velasco-Mondragón E (2011). Self-collection of vaginal specimens for human papillomavirus testing in cervical cancer prevention (MARCH): a community-based randomised controlled trial. Lancet.

[30] Arbyn M, Sankaranarayanan R, Muwonge R, Keita N, Dolo A, Mbalawa CG (2008). Pooled analysis of the accuracy of five cervical cancer screening tests assessed in eleven studies in Africa and India. Int J Cancer.

[31] Elit L, Baigal G, Tan J, Munkhtaivan A (2006). Assessment of 2 cervical screening methods in Mongolia: cervical cytology and visual inspection with acetic acid. J Low Genit Tract Dis.

[32] De Vuyst H, Claeys P, Njiru S, Muchiri L, Steyaert S, De Sutter P (2005). Comparison of pap smear, visual inspection with acetic acid, human papillomavirus DNA-PCR testing and cervicography. Int J Gynaecol Obstet.

[33] Umulisa MC, Franceschi S, Baussano I, Tenet V, Uwimbabazi M, Rugwizangoga B (2018). Evaluation of human-papillomavirus testing and visual inspection for cervical cancer screening in Rwanda. BMC Womens Health.

[34] Suwansura P, Darojn D (2017). Accuracy of cervical visual inspection with acetic acid guide for 4-quadrant random cervical biopsies by general practitioners in women with abnormal pap smears. Asian Pac J Cancer Prev.

[35] Parashari A, Singh V (2013). Reasons for variation in sensitivity and specificity of visual inspection with acetic acid (VIA) for the detection of pre- cancer and cancer lesions of uterine cervix. Asian Pac J Cancer Prev.

[36] Bhatla N, Mukhopadhyay A, Kriplani A, Pandey RM, Gravitt PE, Shah KV (2007). Evaluation of adjunctive tests for cervical cancer screening in low resource settings. Indian J Cancer.

[37] Morales Flores E S. Factores asociados a la realizacion del PAP a las mujeres de la red de salud cercado https://docplayer.es/15281083-Factores-asociados-a-la-realizacion-del-pap-a-las-mujeres-red-de-salud-cercado-cbba.html. Accessed 14 Feb 2019.

[38] Sankaranarayanan R (2014). Screening for cancer in low- and middle-income countries. Ann Glob Health.

[39] Goldie SJ, Gaffikin L, Goldhaber-Fiebert JD, Gordillo-Tobar A, Levin C, Mahé C, et al. Cost-Effectiveness of Cervical-Cancer Screening in Five Developing Countries. http://dx.doi.org/10.1056/NEJMsa044278. 2009. doi:10.1056/NEJMsa044278. 10.1056/NEJMsa044278 16291985

[40] Dijkstra MG, van Zummeren M, Rozendaal L, van Kemenade FJ, Helmerhorst TJM, Snijders PJF (2016). Safety of extending screening intervals beyond five years in cervical screening programmes with testing for high risk human papillomavirus: 14 year follow-up of population based randomised cohort in the Netherlands. BMJ.

[41] Dexeus S, Cararach M, Dexeus D (2002). The role of colposcopy in modern gynecology. Eur J Gynaecol Oncol.

[42] Abolafia-Cañete B, Monserrat-Jordán JÁ, Cuevas-Cruces J, Arjona-Berral JE (2018). Early diagnosis of cervix cancer: correlation between cytology, colposcopy and biopsy. Rev Esp Patol.

[43] Marujo AT, Correia L, Brito M, Paula T, Borrego J (2017). ASC-H cytological result: clinical relevance and accuracy of colposcopy in predicting high-grade histological lesions-a 7-year experience of a single institution in Portugal. J Am Soc Cytopathol.

[44] Massad LS, Collins YC (2003). Strength of correlations between colposcopic impression and biopsy histology. Gynecol Oncol.

[45] Wang M, Hu S, Zhao S, Zhang W, Pan Q, Zhang X (2017). Accuracy of triage strategies for human papillomavirus DNA-positive women in low-resource settings: a cross-sectional study in China. Chin J Cancer Res.

[46] Blumenthal PD, Gaffikin L, Chirenje ZM, McGrath J, Womack S, Shah K (2001). Adjunctive testing for cervical cancer in low resource settings with visual inspection, HPV, and the pap smear. Int J Gynaecol Obstet.

[47] Purwoto G, Dianika HD, Putra A, Purbadi S, Nuranna L (2017). Modified Cervicography and visual inspection with acetic acid as an alternative screening method for cervical precancerous lesions. J Cancer Prev.

