# Supplementary material for: Evaluation of the effectiveness of high-risk human papilloma self-sampling test for cervical cancer screening in Bolivia
Source: BMC Infect Dis. 2020 Apr 3;20:259. doi: 10.1186/s12879-020-04963-2 (PMC7119273; doi:10.1186/s12879-020-04963-2)
Supplement: Supplementary file 1 — Additional file 1. Bolivian anatomo-pathology analyses, by rereading 29 biopsies out of 101 biopsies chosen at random in a Belgian laboratory. [file 12879_2020_4963_MOESM1_ESM.pdf]

Biopsy result agreement to detect CIN2 or worse

	Rereading biopsy results in Belgium		
	Agreement N (%)	Disagreement N (%)	Total biopsies
Bolivian biopsies result	16 (84 %)	3 (16 %)	19 (100 %)

Biopsy result agreement to detect CIN1

	Rereading biopsy results in Belgium		
	Agreement N (%)	Disagreement N (%)	Total biopsies
Bolivian biopsies result	7 (70 %)	3 (30 %)	10 (100 %)
